# Increased susceptibility to bladder inflammation in smokers: targeting the PAF–PAF receptor interaction to manage inflammatory cell recruitment

**DOI:** 10.14814/phy2.12641

**Published:** 2015-12-10

**Authors:** John Marentette, Grant Kolar, Jane McHowat

**Affiliations:** ^1^ Department of PathologySaint Louis University School of MedicineSt. LouisMissouri

**Keywords:** Endothelium, inflammation, interstitial cystitis, smoking

## Abstract

Chronic bladder inflammation can result in a significant reduction in quality of life. Smoking remains a leading preventable risk factor in many diseases. Despite the large amount of evidence supporting the risks of smoking, roughly 45 million people in the United States remain smokers. The impact of cigarette smoking on inflammation is well established, but how smoking promotes bladder inflammation is currently unknown. The aim of this study was to determine if cigarette smoke exposure impacts inflammatory cell adherence to bladder endothelial cells and if targeting the platelet‐activating factor (PAF)–PAF receptor (PAFR) interaction could be beneficial in managing bladder inflammation. In response to cigarette smoke extract (CSE) incubation, bladder endothelial cells from human or mouse displayed increased PAF accumulation, decreased PAF‐AH activity, and increased inflammatory cell adherence. Inhibition of endothelial cell calcium‐independent phospholipase A_2_ *β* (iPLA_2_ *β*) with *(S)*‐BEL, to block PAF production, prevented adherence of inflammatory cells. Pretreatment of inflammatory cells with PAFR antagonists, ginkgolide B or WEB2086 significantly reduced the number of adhered cells to bladder endothelium. Wild‐type mice exposed to cigarette smoke displayed increased presence of inflammatory infiltration which was absent in iPLA_2_ *β* ^−/−^ mice and those exposed to room air. In conclusion, cigarette smoke exposure increases endothelial cell PAF accumulation and increased inflammatory cell adherence. Inhibition of PAF accumulation or PAFR antagonism markedly attenuated inflammatory cell adherence to bladder endothelial cells. The results detailed in this study highlight to potential therapeutic targets for managing bladder inflammation.

## Introduction

Inflammatory bladder diseases, such as interstitial cystitis/bladder pain syndrome (IC/BPS) and urinary tract infections (UTI) can result in a patient's quality of life being significantly reduced (Warren et al. 2008; Clemens et al. 2011). Migration of inflammatory cells from the circulation is a multistep process that starts with the tethering of an inflammatory cell to the apical endothelial cell surface. This is followed by inflammatory cells rolling across the endothelium, adhering tightly and finally transmigration between neighboring endothelial cells facilitated by adhesion molecules and platelet‐activating factor (PAF) (Green et al. 2004; Rastogi et al. 2008). Generation of endothelial cell PAF is initiated by calcium‐independent phospholipase A_2_ *β* (iPLA_2_ *β*) activation, which hydrolyzes the *sn*‐2 fatty acid from membrane phospholipids, yielding a free fatty acid and lysophospholipid. The lysophospholipid, 1‐*O*‐alkyl‐sn‐glycero‐3‐phosphocholine, is subsequently acetylated at the *sn*‐2 position, yielding PAF (McHowat et al. 2001; Beckett and McHowat 2008). Increased endothelial cell surface accumulation of PAF leads to inflammatory cell adherence and transendothelial migration, via binding to the inflammatory cell PAF‐receptor (PAFR) (Sharma et al. 2014). PAF is normally maintained at low concentrations by hydrolysis by PAF‐acetylhydrolase (PAF‐AH), which removes the acetyl group from the *sn‐*2 position yielding the inactive lyso‐PAF.

Smoking remains a leading preventable risk factor in many diseases. Despite the large amount of evidence supporting the risks of smoking, roughly 45 million people in the United States remain smokers (Kandel et al. 2011; King et al. 2012) with an even greater number that are regularly exposed to secondhand smoke, an estimated 126 million including children (Max et al. 2012). In 1989, it was first noted that PAF levels in serum were higher in smokers than in nonsmokers (Imaizumi et al. 1990, 1991; Narahara and Johnston 1993). Cigarette smoking is the primary risk factor for the development of bladder cancer (Sosnowski and Przewoźniak 2015) and the impact of smoking on promoting inflammation has been well established. To date, the impact of cigarette smoking on inducing bladder inflammation is not known. Smoking cessation is recommended for patients with bladder conditions as many components of cigarette smoke can accumulate and concentrate in the bladder. This study aims to determine the impact of cigarette smoke exposure on promoting bladder inflammation. In this study, we show that cigarette smoke extract (CSE) increases inflammatory cell adherence to the bladder endothelium via increased PAF accumulation. Furthermore, we show that blocking the PAF–PAFR interaction with the *Ginkgo biloba* extract, ginkgolide B, inhibits inflammatory cell adherence and thus may be beneficial in the management of bladder inflammation. This data shows that cigarette smoke increases the potential for bladder inflammation, which could be a precipitating factor for the development of inflammatory bladder conditions.

## Materials and Methods

### Bladder endothelial cell culture

Human bladder microvascular endothelial cells (HBMEC) were grown in EGM‐2MV medium (Lonza, Walkersville, MD) and maintained at 37°C in a humidified atmosphere of 95% O_2_ and 5% CO_2_. Cells were treated with cigarette smoke extract (CSE, 20 μg/mL) for indicated times as previously described (Sharma et al. 2012). CSE was obtained from Murty Pharmaceuticals (Lexington, KY).

### Mouse bladder endothelial cell isolation

Animal protocols were in strict accordance with the National Institutes of Health guidelines for humane treatment of animals, and were reviewed and approved by the Animal Care and Use Committee of Saint Louis University. Endothelial cells were isolated from mouse bladder by collagenase digestion. The diced bladder was digested in 1 mg/mL collagenase for 1 h at 37°C. Cells were incubated with murine immunoglobulins to block Fc receptors and then incubated with anti‐mouse platelet endothelial cell adhesion molecule‐1 (PECAM‐1) coupled to magnetic beads. The eluted cells were washed, resuspended in cell culture medium, and plated. Nonadherent cells were removed the next day, and cells were grown to confluence and passaged at a 1 in 3 dilution. Isolation purity was verified by staining with anti‐factor VIII antibody and preparations with greater than 85% endothelial purity were used.

### ELISA measurement of PAF accumulation

PAF was measured directly using an ELISA kit (Biotang, Waltham, MA). HBMEC monolayers were washed with ice‐cold Dulbecco's phosphate‐buffered saline (D‐PBS) and frozen at −20°C. After two freeze‐thaw cycles, aliquots of the suspension were added to microtiter plates with a biotin‐conjugated polyclonal antibody specific for PAF. PAF content in samples was determined spectrophotometrically at 450 nm using a Synergy 2 microplate reader (Biotek, Winooski, VT).

### Radiometric assay for PAF production

Endothelial cells grown to confluence were incubated with Hanks’ balanced salt solution containing 10 μCi of [^3^H] acetic acid for 20 min at room temperature. Total lipid extracts were resuspended in 9:1 CHCl_3_:MeOH and applied to TLC plates. Plates were developed in 100:50:16:8 chloroform, methanol, acetic acid, and water. The region corresponding to PAF was scraped and measured by liquid scintillation counting.

### Measurement of PAF‐AH activity

Endothelial cells were grown to confluence, harvested in 1.2 mmol/L Ca^2+^ HEPES buffer, and sonicated on ice. Cellular protein (25 μg) was incubated with 0.1 mmol/L [acetyl‐^3^H] PAF (10 mCi/mmol) for 30 min at 37°C. The reaction was stopped by adding 50 μL 10 mol/L acetic acid and 1.5 mL 0.1 mol/L sodium acetate. Released [^3^H]acetic acid was isolated by passing the reaction mixture through a C_18_ gel cartridge (Baker Chemical Co., Phillipsburg, NJ) and radioactivity was measured using a liquid scintillation counter.

### Measurement of PMN adherence

Human PMN were isolated from peripheral blood and separated from red blood cells following centrifugation. PMN (2 × 10^6^) added to HBMEC grown to confluence in 34‐mm dishes. At the end of incubation, nonadherent cells were removed, and then HBMEC and adherent PMN were lysed with 0.2% Triton X‐100 and myeloperoxidase (MPO) content was determined by adding 400 μL of cell lysate to a tube containing 1 mL of PBS, 1.2 mL Hanks buffer with bovine serum albumin, 200 μL of 0.125% 3,3′‐dimethoxybenzidine, and 200 μL of 0.05% H_2_O_2_. After samples were incubated at 37°C for 15 min, the reaction was stopped by the addition of 200 μL of NaN_3_, and the absorbance was measured at 460 nm. MPO content in 2 × 10^6^ PMN was determined and used as the value for 100% adherence. In selected experiments, PAFR antagonists, WEB 2086 and ginkgolide B, were added to PMN (10 μmol/L, 30 min) prior to addition to endothelial cells.

### Measurement of RAW 246.7 adherence

RAW 264.7 cells were grown to confluence in Dulbecco's modified Eagle's medium with 10% fetal bovine serum. Cell suspensions (10 × 10^6^/mL) were labeled with 4 μg/mL calcein‐AM for 45 min at 37°C. Cells were washed three times with HEPES buffer and resuspended at a concentration of 4 × 10^6^/mL, and 0.5 mL was added to confluent HBMEC monolayers. Adherent cells and endothelial cells were lysed in 1 mL of 0.2% Triton X‐100. Calcein fluorescence in each sample was measured at an excitation wavelength of 485 nm and an emission wavelength of 530 nm.

### Resistance measurements in bladder endothelial cells

HBMEC, grown to confluence on Transwell inserts, were incubated with CSE (20 μg/mL) or in media alone and changes in electrical resistance were measured over time using an epithelial volt ohmmeter.

### Adhesion molecule cell surface expression

HBMEC, grown to confluence in 16‐mm culture dishes, were incubated with CSE (20 μg/mL) for indicated times at 37°C in 95% O_2_‐5% CO_2_. At the end of incubation, buffer was quickly removed, and cells were immediately fixed with ice‐cold 1% paraformaldehyde and incubated overnight at 4°C. Cells were washed three times with phosphate‐buffered saline (PBS) and then blocked with Tris‐buffered saline‐Tween supplemented with 0.8% BSA (wt/vol) and 0.5% fish gelatin (wt/vol) for 1 h at 24°C. Appropriate primary antibody (1:50) was used before treatment with horseradish peroxidase‐conjugated secondary antibody (1:5000). Subsequently, each well was incubated in the dark with the 3,3′,5,5′‐tetramethylbenzidine liquid substrate system. Reactions were stopped by the addition of sulfuric acid, and color development was measured with a microtiter plate spectrophotometer at 450 nm.

### In vivo cigarette smoke exposure

Mice were exposed to cigarette smoke generated from the University of Kentucky 3R4F research cigarette with the SciReq InExpose system (Montreal, QC, Canada) using the Federal Trade Commission/International Standard Organization standard of 35‐mL puffs of 2 sec duration taken once a minute. Mice were exposed to cigarettes smoke for 48 min/day, 5 days/week for 4 weeks.

### In vivo lymphoid aggregate analysis

Inflammation was determined in a blinded fashion and represented as the average number of lymphoid aggregates per animal group. Lymphoid aggregates were defined as large collections of lymphocytes within the submucosa located just beneath the epithelium (often with thinning of the overlying epithelium) and away from the vasculature. Aggregates were typically twice as large as those located near the base of the submucosa and those associated with vessels, which are considered a normal feature of the bladder mucosa.

### Statistical analysis

All studies were repeated with at least four separate cell cultures. Data were analyzed using Student's *t*‐test or one‐way analysis of variance followed by post hoc analysis using Dunnett's test. Differences were regarded as significant at *P *<* *0.05 and highly significant at *P *<* *0.01. Data are means ± SE.

## Results

**Figure 1:**
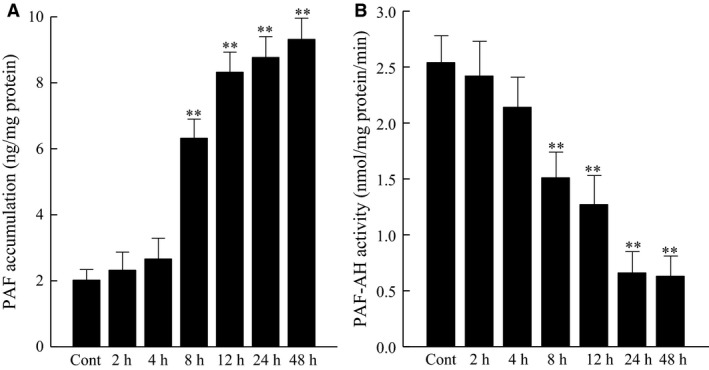
Human bladder microvascular endothelial cells exposed to CSE (20 μg/mL). (A) PAF accumulation. (B) PAF‐AH Activity. *P < 0.05, **P < 0.01 when compared to control. *n* = 4. Data are means ± SE.

To determine the effect of cigarette smoke exposure on bladder inflammation, we incubated HBMEC with CSE (20 μg/mL). We detected a significant increase in HBMEC PAF accumulation after 8 hours of CSE, which continued to increase over time (Fig. 1A). Following incubation of HBMEC with CSE (20 μg/mL), we detected significantly reduced PAF‐AH activity after 8 h, which continued to be reduced as time of CSE exposure increased (Fig. 1B). The reduction in PAF‐AH activity corresponds to the increase in PAF production (Fig. 1A).

**Figure 2:**
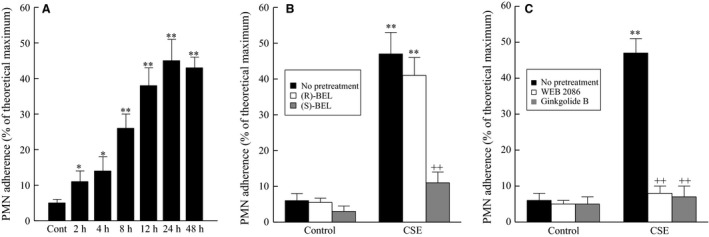
PMN Adherence to human bladder microvascular endothelial cells exposed to CSE (20 μg/mL). (A) PMN Adherence. (B) PMN adherence with iPLA_2_ inhibitors. ^++^ *P* < 0.01 when comparing data in the presence and absence of iPLA_2_ inhibitors. (C) PMN adherence with PAF receptor antagonists. *P < 0.05, **P < 0.01 when compared to control. ^++^ *P* < 0.01 when comparing data in the presence and absence of PAF receptor antagonists. *n* = 4. Data are means ± SE.

We have previously shown that inhibition of HBMEC PAF‐AH with methyl arachidonyl fluorophosphonate (MAFP) leads to increased polymorphonuclear leukocyte (PMN) adherence (Vinson et al. 2005). To determine the role of cigarette smoke exposure on adherence of inflammatory cells to HBMEC, we added PMN (2 × 10^6^) to confluent HBMEC monolayers exposed to CSE (20 μg/mL) and measured adherence. We observed increased adherence of PMN to the endothelial monolayer after two hours of CSE exposure that was greater after 8 h (Fig. 2A). This data suggests that cigarette smoking increases the adherence of circulating inflammatory cells to the bladder endothelium earlier than at times where we observed CSE‐induced PAF‐AH activity inhibition and increasing PAF accumulation.

The production of endothelial cell PAF is mediated by the activity of calcium‐independent phospholipase A_2_ *β* (iPLA_2_ *β*) (Sharma et al. 2010, 2011). In response to CSE, we observed a significant increase in inflammatory cell adherence to bladder endothelial cells compared to controls (Fig. 2A) and that pretreatment with the iPLA_2_ *γ*‐specific inhibitor (*R*)‐BEL had no significant effect on PMN adherence (Fig. 2B). However, when iPLA_2_ *β* activity is inhibited with (*S*)‐BEL there is a significant reduction in PMN adherence even in the presence of CSE (Fig. 2B). In Fig. 2C, we show that pretreatment of the inflammatory cells with a synthetic PAF receptor antagonist (WEB 4086, 10 μmol/L) or the by use of an extract of the *Ginkgo biloba* plant, ginkgolide B (10 μmol/L), reduces inflammatory cell adherence to control levels even when the endothelial cells are exposed to CSE.

**Figure 3:**
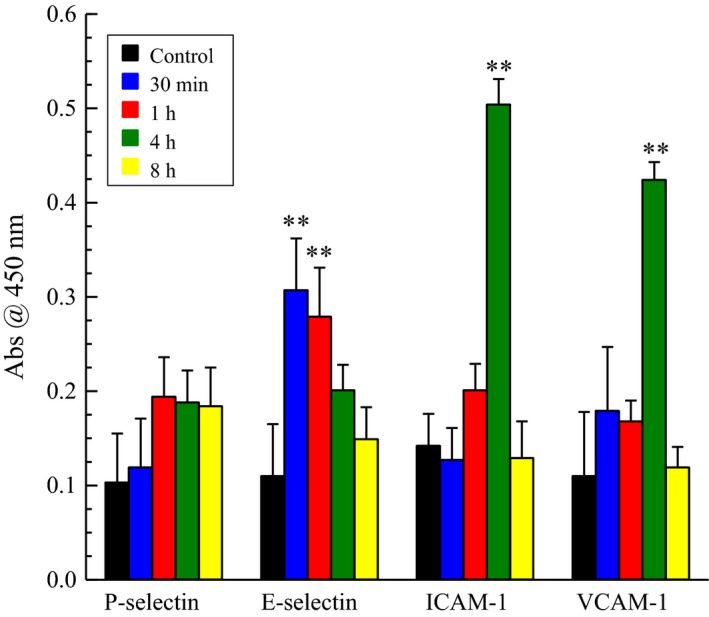
Cell surface expression of adhesion molecule in human bladder microvascular endothelial cells exposure to CSE (20 μg/mL). **P < 0.01 when compared to control. *n* = 8.

As shown in Figure 2A, we measured adherence of PMN to HBMEC following 2 h of CSE, but there was no detectable PAF accumulation until after 8 h of CSE exposure. To account for the adherence seen following 2 h of CSE exposure, we measured the cell surface expression of cell adhesion molecules known to be involved in inflammatory cell adherence, such as P‐selectin, E‐selectin, ICAM‐1, and VCAM‐1. As shown in Figure 3, we detected significant increases in E‐selectin following 30 min to 1 h of CSE exposure, which is lost by 4 h. After 4 h of CSE exposure, we saw a significant increase in both ICAM‐1 and VCAM‐1 expression, which returned to controls levels by 8 h. The observed increase in adhesion molecule expression in HBMEC occurred more rapidly in response to CSE incubation than has been previously noted (Dvorin et al. 2003) following cytokine stimulation of endothelial cells from major vessels. Despite the increases in cell surface expression of these adhesion molecules, blocking the PAF receptor with ginkgolide B‐inhibited PMN adherence at any time of CSE exposure studied (data not shown).

**Figure 4:**
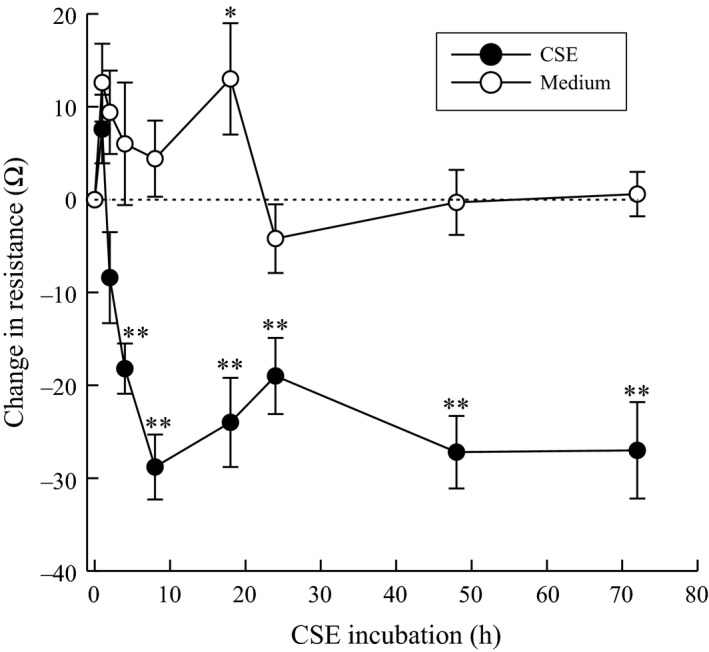
Change in electrical resistance of human bladder microvascular endothelial cells exposed to CSE (20 μg/mL). **P < 0.01 when compared to control. *n* = 5.

To determine the effect of cigarette smoke exposure on vascular endothelial permeability, we measured the change in electrical resistance across the endothelial monolayer with or without CSE exposure. As shown in Figure 4, we detected a significant decrease in electrical resistance in endothelial cells following exposure to CSE compared to those grown in media. The observed reduction in electrical resistance remained persistent over the course of the experimental time. This observed change is indicative of increased vascular permeability, which could result in a greater flux of inflammatory cells from circulation into the underlying tissue increasing inflammation and pain.

**Figure 5:**
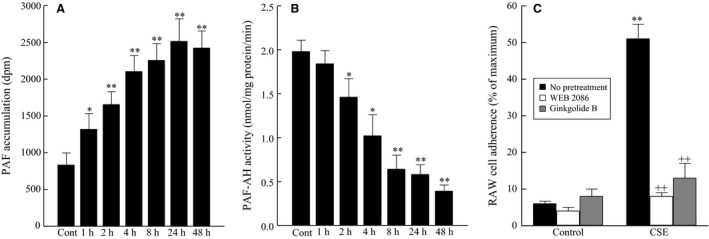
Mouse bladder endothelial cells exposed to CSE (20 μg/mL). (A) PAF accumulation. (B) PAF‐AH Activity. (C) RAW 246.7 cell adherence with PAF receptor antagonists. *P < 0.05, **P < 0.01 when compared to control. ^++^ *P* < 0.01 when comparing data in the presence and absence of PAF receptor antagonists. *n* = 6. Data are means ± SE.

Incubation of isolated bladder endothelial cells from wild‐type mice with CSE resulted in a significant increase in PAF accumulation, similar to that observed with HBMEC (Fig. 5A). The activity of PAF‐AH (Fig. 5B) was reduced in a similar fashion to that observed in HBMEC and corresponds with increased PAF accumulation. Following CSE exposure we incubated mouse bladder endothelial cells with RAW 264.7 cells and measured adherence. We observed significant adherence of RAW 246.7 cells to the mouse bladder endothelial cells compared to control (Fig. 5C). This adherence was blocked with pretreatment of RAW 246.7 cells with either synthetic (WEB 2086) or natural (ginkgolide B) PAFR antagonists, which further highlights the benefit of PAFR antagonism for management of bladder inflammation.

**Figure 6:**
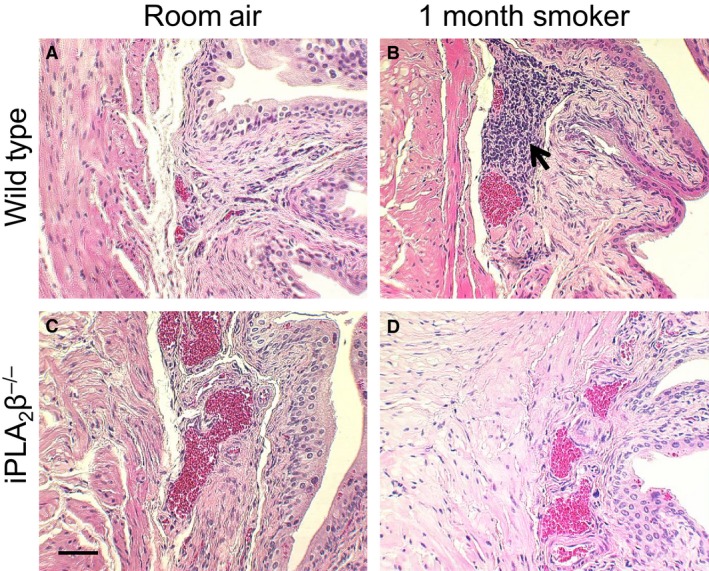
Bladder inflammation following smoke exposure. (A&C) Room Air. (B&D) 4 Weeks of Smoking. (A&B) Wild Type. (C&D) iPLA_2_ *β* ^−/−^. *n* = 5–8. Arrow = Inflammatory Cells. Representative Images. Scale Bar = 20 μm.

**Figure 7:**
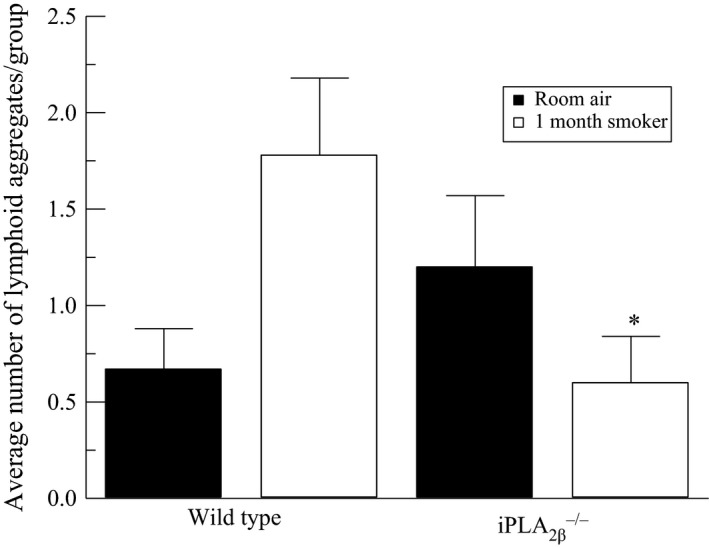
Average number of lymphoid aggregates per animal group following 4 weeks of cigarette smoke exposure. *P < 0.05 when compared to wild‐type counterpart. *n* = 5–8.

To further explore the effect of cigarette smoke exposure on bladder inflammation, we exposed mice to smoke generated from the University of Kentucky 3R4F research cigarettes for 4 weeks. We detected the presence of inflammatory cells in the bladder wall of wild‐type mice exposed to cigarette smoke compared to room air (Fig. 6). When compared to wild‐type mice, iPLA_2_ *β* ^−/−^ mice exposed to cigarette smoke showed minimal inflammatory cell infiltration (Fig. 6). As shown in Figure 7, following 4 weeks of cigarette smoke exposure, we observed an increase in the average number of lymphoid aggregates in the bladder wall compared to room air animals. In the iPLA_2_ *β* ^−/−^ mice, we observed a significant reduction in the number of lymphoid aggregates compared to the wild‐type mice exposed to cigarette smoke. This data suggests that a lack of iPLA_2_ *β* and therefore inhibited PAF production could attenuate the inflammatory response in the bladder wall.

## Discussion

Bladder diseases can significantly impact the quality of life in those who suffer from them. Commons disorders of the bladder include overactive bladder syndrome (OAB), urinary tract infection (UTI), interstitial cystitis/bladder pain syndrome (IC/BPS), and bladder cancer. The common symptom amongst these conditions, increased urinary frequency, can alone have a significant impact on quality of life and ability to perform normal daily activities (Teichman and Parsons 2007; Warren et al. 2008; Clemens et al. 2011). Pain and inflammation are a common characteristic in UTI and IC/BPS, which can be debilitating. UTI are managed by administration of antibiotics, but treatment options for IC/BPS are limited and lack significant efficacy. Patients with a history of UTI have been shown to be more prone to developing subsequent infections. It is not uncommon for patients with IC/BPS to have a UTI at onset of IC/BPS symptoms and it has been proposed that UTI might be an acute trigger to the chronic inflammatory condition of IC/BPS in some patients (Warren et al. 2008).

Mast cells have long been known to be involved in chronic inflammatory responses, such as those seen in IC/BPS and UTIs, and have recently been shown to be increased in higher grade bladder tumors (Kim et al. 2011; Chuang and Kuo 2013). Mast cells have been shown to increase the expression of cellular adhesion molecules and bladder tissue samples from IC/BPS patients show expression of adhesion molecules including P‐selectin, E‐selectin, and ICAM‐1 compared to control tissue (Green et al. 2004). We have previously shown that tryptase, a mast cell released protease, increases activity of calcium‐independent phospholipase A_2_ (iPLA_2_) in bladder endothelial cells (Rickard et al. 2005). Mast cells express the PAF‐R (Rastogi et al. 2008) and therefore, mast cell‐stimulated increases in endothelial cell PAF could prolong and propagate the mast cell‐associated inflammatory response leading to chronic inflammation. Our data illustrate a potential link between cigarette smoking and bladder inflammation mediated by increased PAF accumulation and recruitment of inflammatory cells to the endothelium, which is supported by previous studies (Lehr et al. 1997). We have demonstrated previously that CD133+ cells, a mast cell precursor population, utilizes the PAF–PAFR interaction in a manner similar to that involved in PMN transendothelial migration (Rastogi et al. 2008). Therefore, we propose that increased endothelial cell PAF production in smokers may result in increased recruitment and accumulation of mast cells in the bladder wall. In the bladder wall, activation of mast cells and subsequent release of granular contents can stimulate substance P release from unmyelinated nerve fibers causing further mast cell activation and increased bladder pain.

Focusing therapeutic interventions toward the endothelium could provide a promising approach for the management of bladder inflammation. In the experiments outlined above, we show that modulation of PAF production could have exciting therapeutic potential for management of bladder inflammation. The in vitro data presented above demonstrates that exposure to cigarette smoke‐derived components leads to the increased potential for developing bladder inflammation by inhibiting PAF‐AH activity leading to increased endothelial PAF accumulation. The inverse relationship between PAF‐AH activity and PAF accumulation has been previously noted (Chen et al. 2007). Inhibition of iPLA_2_ *β* with *(S)*‐BEL blocks inflammatory cell adherence. This in vitro data is further supported by the observations that iPLA_2_ *β* ^−/−^ mice show significantly reduced bladder inflammation compared to wild‐type mice. These experiments highlight the importance of PAF production in the inflammatory response in the bladder. Therefore, in chronic inflammatory conditions, modulation of PAF could be a promising avenue to exploit for inflammation management. *Ginkgo biloba* is a nutraceutical used in the management of several diseases including asthma, circulatory diseases, and memory loss (Braquet 1986; Oberpichler et al. 1990; Braquet et al. 1991; Grypioti et al. 2008). Extracted from *Ginkgo biloba,* ginkgolide B is excreted unchanged in the urine making it an ideal therapeutic intervention for managing bladder inflammation (Kimbel 1992). We show that pretreatment of inflammatory cells with PAF‐R antagonists, such as those in Ginkgo biloba extracts, significantly inhibited the adherence of inflammatory cells to human and mouse bladder endothelial cells. This inhibition occurred even in the presence of enhanced PAF accumulation.

One limitation to the adherence studies highlighted is the use of confluent monolayers as our culture system. Confluent endothelial monolayers in vitro express few cytoplasmic vesicles and lack the underlying cells that typically modify endothelial cell behavior resulting in a “leakier” phenotype compared to endothelial cells in vivo (Nagy et al. 2012). However, in these in vitro studies, we are measuring adherence to endothelial monolayers as opposed to transendothelial migration, which combined with our results obtained in vivo support our hypothesis that cigarette smoking contributes to bladder inflammation via increased PAF production. We have shown in vitro using human and mouse bladder endothelial cells that PAF accumulation occurs in response to CSE exposure and that as PAF increases, so does inflammatory cell adherence. After exposing wild‐type mice to cigarette smoke for 4 weeks, we detected inflammatory cell infiltration in the bladder wall. To determine if the observed inflammatory cell infiltration may be due to PAF accumulation, we exposed iPLA_2_ *β* ^−/−^ mice to cigarette smoke in the same manner as the wild‐type. Based on our hypothesis that PAF is mediating the observed inflammation, the lack of iPLA_2_ *β* and therefore a lack of endothelial cell PAF production would reduce inflammatory cell infiltration. In the iPLA_2_ *β* ^−/−^ mouse, substantially reduced inflammatory cell infiltration was an exciting finding that further supports the role of iPLA_2_ and PAF in the establishment of an inflammatory response in the bladder. Further experimentation needs to be performed to determine if oral administration of PAFR antagonists would reduce bladder inflammation in the presence of cigarette smoking. The experiments outlined above provide evidence for the use of naturally occurring PAFR agonists for the management of bladder inflammation.

## Conclusions

Exposure to cigarette smoke increases the susceptibility for developing bladder inflammation by inhibiting the activity of PAF‐AH and increasing accumulation of endothelial cell PAF. The interaction between endothelial cell PAF and inflammatory cell PAFR can be inhibited by administration of PAFR antagonists, such as WEB2086 or ginkgolide B, which has a significant impact on the ability of inflammatory cells to transmigrate the endothelium.

## Conflict of Interest

None declared.
